# Supplementary figures and images for: m6A modification of U6 snRNA modulates usage of two major classes of pre-mRNA 5’ splice site
Source: eLife. 2022 Nov 21;11:e78808. doi: 10.7554/eLife.78808 (PMC9803359; doi:10.7554/eLife.78808)

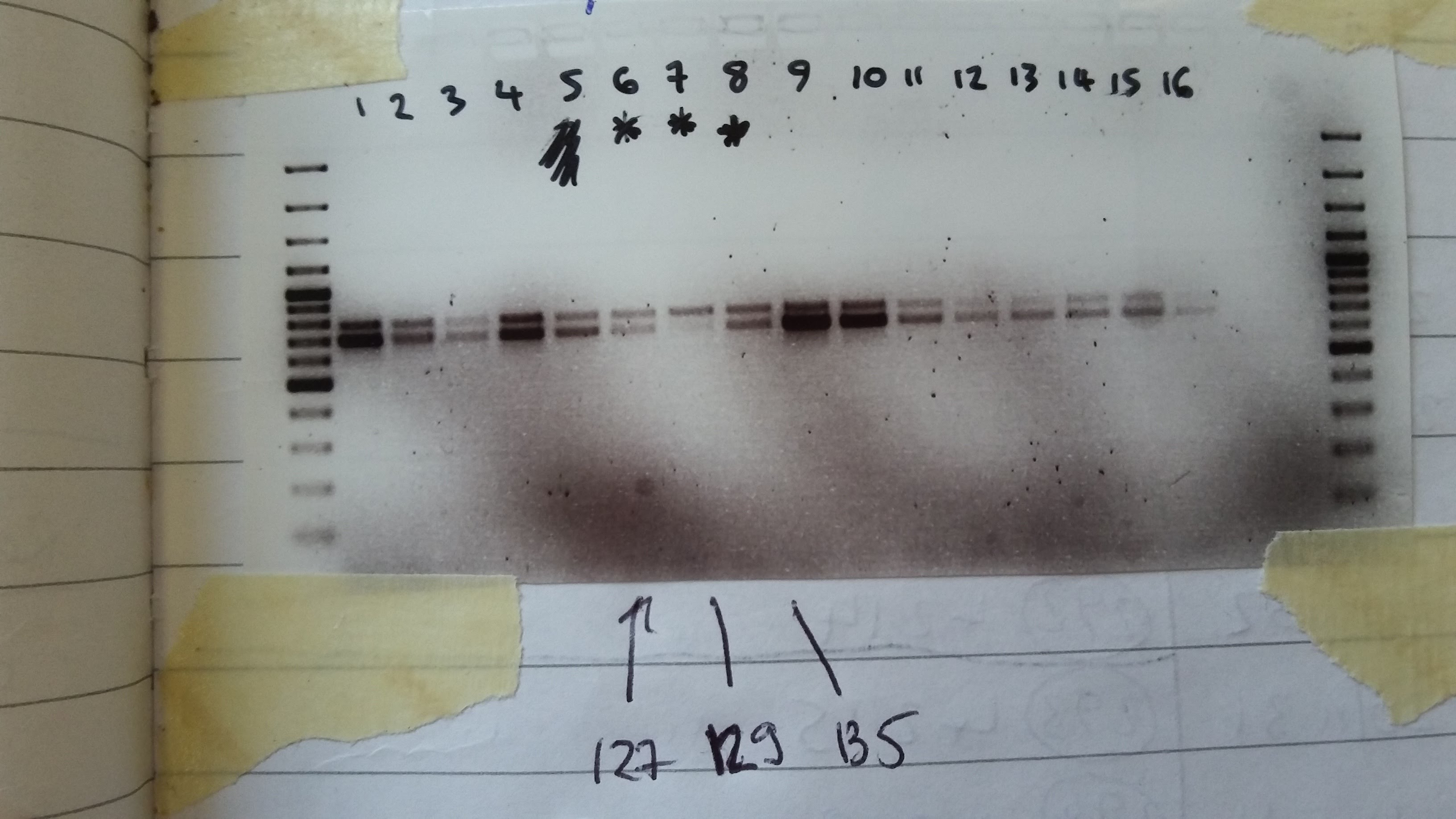

Supplement: Figure 1—source data 6. [file elife-78808-fig1-data6.zip › figure_1_source_data_6.jpeg]
